# The Norwalk Catastrophe—Meeting of the Medical Association

**Published:** 1853-06

**Authors:** 


THE NORWALK CATASTROPHE—MEETING OF THE MEDICAL ASSOCIATION.

A meeting of delegates to the late Medical Convention, still remaining in New York, was held yesterday morning at the Bleecker Street Church, corner of Bleecker and Crosby Streets, for the purpose of expressing the feelings of the Association on the loss of several members of that body by the railway catastrophe at Norwalk. Dr. Joseph M. Smith was appointed President of the meeting, and Dr. E. L. Beadle officiated as Secretary. On motion of Dr. Rockwell, a Committee was appointed to prepare suitable resolutions for the occasion. The following gentlemen formed the Committee: J. H. Griscom, N. Y.; S. Hanbury Smith, Ohio; P. Claiborne Gooch, Va.; L. A. Smith, N. J.; Theodore Goodloe, Ala.; R. La Roche, Pa.; John Watson, N. Y.

The following preamble and resolutions were read by J. H. Griscom, M. D.

*Whereas*, amid the wide-spread affliction caused by the recent catastrophe at Norwalk, the members of the American Medical Association, recognizing in this mournful event the hand of an all-wise Providence, feel called upon to express their grief at the sudden removal from life of Abel L. Pierson, M. D., of Salem, Mass; Alexander Welch, M. D., of Hartford, Conn.; Josiah Bartlett, M. D., of Stratham, N. H.; Samuel Beach, M. D., of Bridgeport, Conn.; James M. Smith, M. D., and J.

H. Gray, M. D., of Springfield, Mass., late members of the Association:* And, *whereas,* it is the earnest desire of the members, still present in the City of New York, to record a suitable expression of their feelings upon an occasion equally unprecedented and distressing; therefore,

*Resolved,* That the members have received with profound sorrow the lamentable intelligence of the loss which the community, as well as the profession, have sustained, by the death of so large a number of the American Medical Association.

*Resolved,* That as a suitable, though inadequate, external mark of their grief at the sudden demise of friends from whom they had so recently parted, the members of the Association in general are recommended to wear the usual badge of mourning for the space of thirty days.

*Resolved,* That a Committee of five be appointed to devise some suitable method of commemorating the event, and the worth and professional character of our lamented associates, and recommend their plan at the next meeting of the Association.

*Resolved,* That the members of the Association deeply sympathize with the relatives of the deceased, and that a copy of these resolutions, duly authenticated, be transmitted to their respective families.

The resolutions were adopted by the meeting, and a Committee was appointed to consider the best method of providing some memorial in commemoration of the terrible disaster. The names of this Committee were Joseph M. Smith, M. D., F. C. Stewart, M. D., J. W. G. Clements, M. D., W. Rockwell, M. D., Isaac E. Taylor, M. D., E. L. Beadle, M. D. and John Watson, M. D.

The report of this Committee will be delivered at the next meeting of the Medical Association, which will take place next year, in St. Louis. —*N. J. Med. Reporter.*

* Among the killed was Dr. Wm. C. Dwight, of Brooklyn, N. Y., who was probably not a member of the Association.—*Ed. N. J. Medical Reporter.*

